# Characterization of Epileptic Spiking Associated With Brain Amyloidosis in APP/PS1 Mice

**DOI:** 10.3389/fneur.2019.01151

**Published:** 2019-11-12

**Authors:** Irina Gureviciene, Irina Ishchenko, Sofya Ziyatdinova, Nanxiang Jin, Arto Lipponen, Kestutis Gurevicius, Heikki Tanila

**Affiliations:** ^1^A.I. Virtanen Institute, University of Eastern Finland, Kuopio, Finland; ^2^Academy of Biology and Biotechnology, Southern Federal University, Rostov-on-Don, Russia; ^3^Department of Psychology, University of Jyväskylä, Jyväskylä, Finland

**Keywords:** Alzheimer's disease, amyloid - beta- protein, transgenic, EEG, sleep, epilepsy, cortex, hippocampus

## Abstract

Epileptic activity without visible convulsions is common in Alzheimer's disease (AD) and may contribute adversely to the disease progress and symptoms. Transgenic mice with amyloid plaque pathology also display epileptic seizures, but those are too infrequent to assess the effect of anti-epileptic treatments. Besides spontaneous seizures, these mice also display frequent epileptic spiking in epidural EEG recordings, and these have provided a means to test potential drug treatment to AD-related epilepsy. However, the origin of EEG spikes in transgenic AD model mice has remained elusive, which makes it difficult to relate electrophysiology with underlying pathology at the cellular and molecular level. Using multiple cortical and subcortical electrodes in freely moving APP/PS1 transgenic mice and their wild-type littermates, we identified several types of epileptic spikes among over 15 800 spikes visible with cortical screw electrodes based on their source localization. Cortical spikes associated with muscle twitches, cortico-hippocampal spikes, and spindle and fast-spindle associated spikes were present equally often in both APP/PS1 and wild-type mice, whereas pure cortical spikes were slightly more common in APP/PS1 mice. In contrast, spike-wave discharges, cortico-hippocampal spikes with after hyperpolarization and giant spikes were seen almost exclusively in APP/PS1 mice but only in a subset of them. Interestingly, different subtypes of spikes responded differently to anti-epileptic drugs ethosuximide and levetiracetam. From the translational point most relevant may be the giant spikes generated in the hippocampus that reached an amplitude up to ± 5 mV in the hippocampal channel. As in AD patients, they occurred exclusively during sleep. Further, we could demonstrate that a high number of giant spikes in APP/PS1 mice predicts seizures. These data show that by only adding a pair of hippocampal deep electrodes and EMG to routine cortical epidural screw electrodes and by taking into account underlying cortical oscillations, one can drastically refine the analysis of cortical spike data. This new approach provides a powerful tool to preclinical testing of potential new treatment options for AD related epilepsy.

## Introduction

Accumulating evidence suggests that epilepsy is an integral part of the pathophysiology of Alzheimer's disease (AD) (1). Despite ~8 times more common than in general age-matched population, epileptic seizures with convulsions are rare in AD patients and not considered a significant clinical problem (2). However, a recent prospective EEG study found subclinical epileptiform activity in more than 40% of AD patients (3). Their presence indicated significantly faster cognitive decline in 5-year follow-up. Further, subclinical epileptic discharges have been suggested to contribute to impaired memory and attention, and especially to cognitive fluctuation in AD patients (4). There is thus an unmet need to find well-tolerated but effective treatment for these epileptic discharges. To this end, a fundamental question is whether AD-related epilepsy has a unique underlying mechanism requiring unconventional treatments.

Recent studies have revealed increased epileptic activity, including spontaneous seizures, also in most transgenic mouse lines with amyloid (5–10) or tau pathology (11). These mice offer a powerful tool to investigate the circuit and molecular mechanisms of epileptic activity associated with AD pathology as well as to test suitable treatments options. However, there are several open questions to be addressed before the models can be fully utilized. First, it is not established yet whether seizures in the mouse models are primary generalized or of focal onset. No study so far has been able to identify the epileptic focus in these mouse models, which prevents linking epileptic activity with local pathology that typically varies greatly between brain regions as in early AD. Second, as in AD patients, typical spontaneous generalized seizures in AD model mice are rare; according to the only systematic study so far, they occur on average once every 3 weeks (6). It is a formidable task to statistically assess the effect of antiepileptic treatments or plan multielectrode studies for localizing the focus of so infrequent events (12). Therefore, much more frequent epileptic spikes of short duration have been used as surrogate markers in anti-epileptic treatment trials in AD model mice. All published studies so far have recorded these spikes on the cortex with epidural electrodes. However, so far these have been poorly characterized. First, there have been highly variable duration and amplitude criteria between studies, as for instance <70 ms, 8 x average baseline (13), <15 ms, 2.5 x average baseline (14), and <100 ms, 2 x average baseline (9). Second, all cortical spikes have been lumped together with the assumption that they reflect similar underlying neuronal and network properties. This is not necessarily the case, since due to the small volume of the mouse brain, voltage changes in a strong subcortical generator such as the hippocampus may be volume conducted and seen as cortical spikes. In addition, cortical spikes often appear as part of a larger type of discrete epileptic activity such as spike-wave discharges, which have a known thalamo-cortical generator (15).

Using multiple intracerebral electrode bundles (“stereo-EEG”) or linear silicone probes, this study aimed at characterizing and localizing the generators of spike discharges seen with routine skull EEG in APPswe/PS1dE9 mice with well-documented seizure occurrence (6). In addition, we tested the response of observed spike types to common anti-epileptic drugs ethosuximide and levetiracetam with known different mechanisms of action to gain more insight into their generation. It was found that only some types of spikes detectable with skull-EEG are actually overrepresented in amyloid plaque forming transgenic mice. Further, the underlying neural circuitry is different for different spike types as well as their response to anti-epileptic drugs.

## Methods

### Animals

The main experiments were performed on 8 male APPswe/PS1dE9 transgenic (APP/PS1) and 6 wild-type (WT) littermate mice. The mean weight of each animal was 27–30 g and the age 5–6 months. In addition, a linear silicone probe was implanted in two male APP/PS1 mice aged 3 months, one APP/PS1 male mouse aged 4.5 months was recorded with cortical and hippocampal electrodes, and one APP/PS1 9-month-old male mouse was recorded with movable tetrodes aimed at the hippocampus.

The APP/PS1 mice carry mouse/human APPswe double point mutations and human presenilin-1 gene with deleted exon 9, cointegrated in the same transgene under the mouse PrP promoter (16). The mice originated from a local colony at University of Eastern Finland, established by breeders from John Hopkins University, Baltimore, MD, USA (generous donation by Dr. D. Borchelt). The mice develop first amyloid plaques around 4 months of age (17). This line was originally maintained in a hybrid C3HeJ x C57BL6/J background, but the mice used in this study were derived from backcrossing to C57BL6/J for 23 generations. After electrode implantations, animals were placed in single standard laboratory cages with water and food *ad libitum*. The experiments were conducted according to the Council of Europe (Directive 86/609) guidelines and approved by the Animal Experiment Board in Finland.

### Electrode Implantation

**Figure 1:**
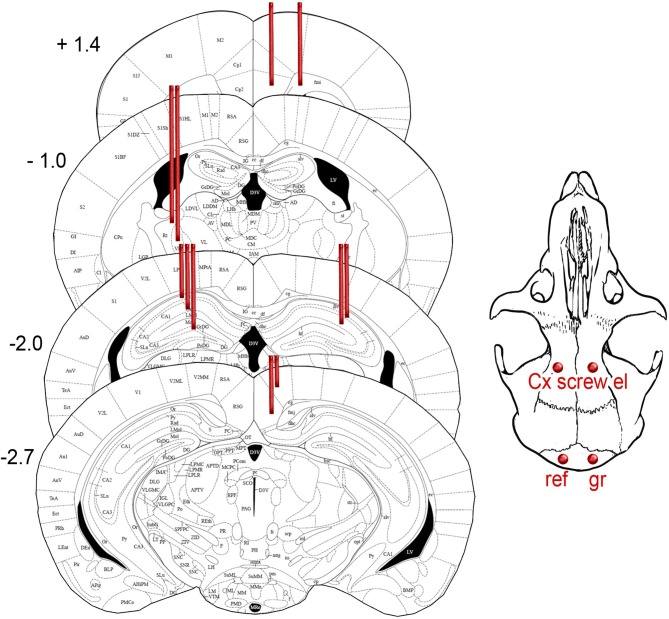
Schematic drawing of the intended location of 15 recording electrodes projected on the closest atlas graphs (18). The coordinates and distance from bregma. The exact location of electrodes in individual mice is summarized in Table 1.

For long-term EEG monitoring mice were implanted with 15 electrodes into the different brain regions (Figure 1). Two screw electrodes (diameter 1.0 mm, length 2.0 mm, Microbiotech/se AB) were fixed bilaterally on the frontal bone at AP 2.7 mm, ML ± 2 mm from bregma. Two parietal screw electrodes were implanted bilaterally on the occipital bone above the cerebellum and were used as ground and reference electrodes. The screws served also as anchors for dental acrylic cement and the connector (Mill-Max, NY, USA). For recording deep brain regions, we implanted wire electrodes (Formwar insulated stainless steel wire, diameter 50 μm, California Fine Wire Company Co, Grover Beach, CA, USA). A pair of wire electrodes were aimed at the medial frontal cortex (AP 1.4, ML 0.4, and 0.9 mm from bregma). Double electrodes with a vertical tip separation of 400 μm were aimed at reticular thalamic nucleus (Th-Rt, AP −1.1, ML −1.7), CA3 layer of hippocampus (AP 2.1, ML +2.4), and retrospenial cortex (RSC, AP −2.9 mm, ML +0.5). A triple wire electrode with a vertical tip separation of 400 μm was implanted in CA1 layer (AP −2.1 mm, ML −1.5). In addition, a polysol-insulated copper wire (diameter 100 μm, Elfa, Finland) was inserted into the neck muscles during surgery for electromyogram (EMG) recording. Two APP/PS1 mice were implanted with a 16-channel linear silicone probe with 100 μm spacing between recording sites (NeuroNexus Technologies, Ann Arbor, MI, USA) into the hippocampus at AP −2.3, ML 1.2 from bregma, so that the active contacts extended through all hippocampal layers. The operation was done under general isoflurane anesthesia (induction 4.5%, maintenance at 1.8–2.1%). After the surgery, the mice received carprofen (5 mg/kg, s.c., Rimadyl®, Vericore, Dundee, UK) for postoperative analgesia, and antibiotic powder (bacitrasin 250 IU/g and neomycinsulfate 5 mg/g, Bacibact, Orion, Finland) was applied. Mice were allowed to recover for 10 ± 3 days before the recording started.

### Recording of EEG and Local Field Potentials

EEG-video recordings were conducted on freely moving animals during the light period in 3-h sessions. During the recordings, the mice were in standard plastic cages (width 18 cm × length 21 cm × height 30 cm) and connected to an 18-ch headstage preamplifier with a light-weighted recording cable (Plexon Inc., Dallas, TX, USA). The signal was further amplified with an AC amplifier (gain 1,000, bandpass-filtering 1–3,000 Hz, Grass, Quincy, MA, USA). The signal was digitized at 2 kHz per channel (DT2821 series A/D board; Data Translation, Marlboro, MA, USA) and acquired by Sciworks 5.0 program (DataWave Technologies, Loveland, CO, USA). Recording for each condition comprised two consecutive 3-h sessions. First, we recorded baseline EEG and continued after a 1-week break with drug treatments. Mice with silicone probes were recorded using Open Ephys 256-ch acquisition board with an A/D converter chip from Intan Technologies (Los Angeles, CA, USA) using Open Ephys GUI software. The data were initially recorded wide-band at 30 kHz per channel and later downsampled to 3 kHz. Mice with tetrodes were also recorded with the Open Ephys system. The tetrodes were twisted from tungsten wire (30 μm, HML insulated, California Fine Wire Company, CA, USA). The tetrodes were loaded into a FlexDrive tetrode carrier (19).

### Assessment of Behavioral State

The behavior of the animals was video recorded with an overhead video camera (Live!Cam, Video IM Pro, Creative, Dublin, Ireland) synchronized with electrophysiological signals. The recordings were analyzed offline using Ethovision (Noldus, Netherlands) video analysis software. Based on the mouse coordinates tracked by Ethovision in every video frame, we smoothened the trajectory to correct unrealistic mouse locations with customized Matlab programs. First, we removed unrealistically sharp turns. Every two subsequently tracked locations make a trajectory vector. If the angle was bigger than 135 degrees or smaller than −135 (meaning the turn to left or right is 45 degrees or smaller), the second point coordinate was corrected as the mean of first and third points. Second, we corrected temporary star-shaped and non-mouse object tracking by comparing the distance from one point to subsequent 4 points. The nearest point of the four was taken as the realistic location. Then the correction continued from the next “nearest point.” Third, we smoothened the trajectory with the Matlab function SMOOTHN using the default parameters (20).

Next, we assigned each 10-s sweep to one of the following behavioral states: movement, waking immobility and sleep. First, instant speeds were calculated between every two neighboring tracking points based on the distance and the time between them. If the instant speed was higher than 0.5 cm/s it was assigned as movement, if lower it was considered immobility. Second, if a single frame with movement was preceded and followed by 10 frames of immobility, if was not considered advancing movement and was also assigned as immobility. Third, immobility periods longer than 30 s were assigned as sleep (i.e., the epoch immediately after 30 s of immobility to the end of this immobility period). The categorization of spikes into sleep, immobility or movement related was based solely on this video tracking.

When assessing the sleep state more closely during the 5 s preceding giant spikes, we used the following criteria. First, video analysis should show immobility and EMG should show a low stable baseline. Second, REM was identified by regular theta oscillation (6–10 Hz) on the hippocampal and retrosplenial channels. Third, NREM was defined by stable large irregular activity on hippocampal channels in general and ripple oscillations when an electrode hit the CA1 layer. When the hippocampal EEG showed short bursts of theta intermingled with large irregular activity it was defined as the transition state.

### Drug Treatments

Ethosuximide (ESM; Sigma-Aldrich, Darmstadt, Germany) was chosen as the prototype drug against absence seizures, and the dose 200 mg/kg was chosen as an effective non-sedative dose (13). Levetiracetam (LEV; Carbosynth Ltd, Campton Bekshire, UK) has become the gold-standard treatment for epileptic seizures and discharges in AD model mice, and dose of 75 mg/kg was estimated to be effective but non-sedative (13). ESM, LEV or normal saline were administered at a volume of 0.10 ml/10 g intraperitoneally 30 min before the recording started. The washout period between treatments was at least 6 days for each animal. The order of drugs was counterbalanced, and the person conducting the recording and analysis was blinded to the treatment.

### Spike Analysis

All signals were analyzed offline in Matlab (Mathworks, Natick, MA, USA; R2015b) using custom-written algorithms. After 50 Hz notch filtering, all channels were high-pass filtered at 8 Hz to remove slow fluctuations from the baseline. Next, the right frontal cortical screw channel was chosen as the channel of interest, and all surface-negative peaks >6 SD below the average filtered baseline and <50 ms in duration were extracted using the MATLAB function “findpeaks” (https://se.mathworks.com/help/signal/ref/findpeaks.html). In addition, all peaks larger than ± 2 SD from the average filtered baseline occurring ± 100 ms of the cortical reference spike were also extracted. Next, an experienced electrophysiologist blinded to the genotype of the mice classified all spikes into one of the following categories based on evaluation of the unfiltered signal.

1. *Cortical spike*; a single spike on one or both cortical screw channels without a hippocampal spike within the ± 100 ms window or EMG activity following the spike.
2. *Cortico-hippocampal spike*; a single cortical spike preceded or followed by a locally generated spike (voltage difference between the 2 and 3 hippocampal channels) within the ±100 ms window in the ipsi- or contralateral hippocampus.
3. *Cortical spike with a muscle twitch*; a type 1 or type 2 spike followed by >200 ms sustained high-frequency activity on the EMG channel.
4. *Spindle-associated spike*; a cortical surface-negative spike initiating a 10–12 Hz spindle oscillation (21) or riding on its trough.
5. *Fast-spindle associated spike*; a cortical surface-negative spike initiating a 12–14 Hz low-amplitude spindle oscillation.
6. *Spike-wave discharge*; an asymmetric complex of at least three cycles of surface-positive 8–10 Hz waves and a sharp surface-negative spikes on either cortical screw channel (22). Each spike in the complex was counted separately.
7. *Cortico-hippocampal spike with after hyperpolarization*; similar to type 2 spike with the exception that in at least two channels the spike is followed by a slow fluctuation of the baseline with suppression of fast activity for at least 200 ms without simultaneous sustained EMG activity.
8. *Giant spike*; a spike resembling type 7 but with four additional criteria: (a) a simultaneous spike (positive or negative) is all channels, (b) a complex of several spikes in different channels resulting in a at least three 0-crossings on the cortical screw channel, (c) massive voltage (>±10 SD from the filtered baseline) in at least one hippocampal channel, (d) after hyperpolarization >200 ms in all channels without simultaneous sustained EMG activity.

### Current Source Density Analysis (CSD)

To identify the source of the giant spikes, we calculated CSD from data recorded with 16-channel linear probes in the hippocampus. First, the giant spikes were detected using the criteria listed above. The maximum peak of any channel was taken as time zero and the current distribution ± 50 ms from that time stamp was calculated. We assumed isotropy of the extracellular space as has been done in previous studies on rodent hippocampus and thus calculated the CSD as the second derivative of potential as a function of depth (23). One of the 16-ch probes has silent channels. To avoid artifactual sinks and sources, we interpolated the voltage in the silent channel as a mean of its neighbors.

### Giant Spikes as Indicator of Seizures

To identify the signature of giant spikes on standard epidural cortical EEG recordings, we first analyzed the voltage deflections of identified giant spikes based on multichannel recordings. In this material derived from 7 APP/PS1 mice, we found that by applying the following criteria the giant spikes could be identified on the cortical screw channel with a sensitivity of 92% and specificity of 91%: (1) amplitude ≥ 650 μV, (2) numbers of zero crossings ≥ 3 during ± 45 ms from the giant spike peak in the hippocampus, (3) occurrence only during immobility. Then we searched the data set of our previous long-term video-EEG recordings on the same APPswe/PS1dE9 line mice of roughly similar age (12, 24, 25). The focus of these studies on the evaluation of the effect of anti-epileptic treatments; however, for this analysis we selected only mice with verified epileptic seizures (usually only one) during pre-treatment baseline recording (Sz+, *n* = 14). Spontaneous seizure was defined as a high amplitude (>2 x baseline) rhythmic discharge that clearly represented an abnormal EEG pattern (repetitive high amplitude spikes, spike-and-wave discharges and slow waves), lasted for ≥ 5 s, and was followed by EEG suppression. We included all seizures irrespective of their severity. As a control, we randomly selected 14 TG mice from the same batch of mice with no documented seizure (Sz -). In this case, without seizure meant that the animal has at least 2 weeks of 24/7 video-EEG recording without a seizure. To compare the general occurrence of giant spikes between Sz+ and Sz- mice, we composed Sz+ and Sz- pairs, so that we analyzed 3 h of EEG data preceding a seizure in the Sz+ mice and compared that to 3 h of EEG at the corresponding time of the day in the Sz- pair.

### Histology

At the end of the experiment, positive DC current was passed through the wire electrodes under deep pentobarbital-chloralhydrate (Equitesin) anesthesia (pentobarbital 50 mg/kg + chloralhydrate 200 mg/kg i.p.). The mouse was perfused through the heart first with ice-cold saline to rinse blood from the cerebral circulation and then with 4% paraformaldehyde solution. Then the brain was removed and immersion fixed for 4 h in 4% paraformaldehyde solution, followed by 30% sucrose overnight. The brain was left in antifreeze at−20°C until cut into 35 μm coronal sections with a freezing slide microtome (Leica). The electrode locations were confirmed in sections stained for glial fibrillary acidic protein (1:1000, Dako, Glostrup, Denmark) to reveal the gliosis around the probes. To visual amyloid plaques, the sections were stained for mouse anti-human antibody WO2 (human anti-amyloid-β, 1:40000, Merck Millipore, Billerica, MA, USA). Sections were incubated overnight at room temperature, treated with a secondary antibody, biotinylated goat anti-mouse (1:1500, Vector Laboratories, Peterborough, UK) and Streptavidin-horseradish peroxidase conjugate (1:1000, GE Healthcare, Buckinghamshire, UK) and visualized by incubation with DAB–Ni solution. Histology revealed that most of the wire electrodes hit the intended location, with the exception of CA3 electrodes that ended up being more medially located, targeting mainly CA1 pyramidal cell layer and dentate hilus. Table 1 summarizes the electrode location in each mouse.

**Table 1:** Location of the electrode tips in individual mice.

**Ch**	**1**	**9**	**10**	**3**	**2**	**12**	**13**	**14**	**5**	**6**	**15**	**16**	**Ref**
Name	FC scr	FC scr	ACC	THs	THd	CA1s	CA1m	CA1d	CA3s	CA3d	RSs	RSd	OC scr
Side	R	L	R	L	L	R	R	R	L	L	R	R	R
**30 tg**	F bone	F bone	Cg1?	TH_LD	TH_Po	CA1p	CA1r	DGg ub	CA1p	hf	RS	cc	O bone
**31 tg**	F bone	F bone	Cg1	fimbria	TH_Rt	CA1o	hf	DGh	CA2p	CA3lu	RS	RS	O bone
**32 wt**	F bone	F bone	M2/Cg1	TH_LD	TH_LD	CA1p	hf	DGh	hf	DGh	RS	RS	O bone
**76 tg**	F bone	F bone	M2/Cg1	TH_LD	TH_Po	hf	DGg ub	DGm lb	CA1r?	DGm ub	V2	cc	O bone
**77 wt**	F bone	F bone	M2	TH_LD	TH_Po	hf	DGg ub	DGm lb	CA1p	CA1r	RS?	RS?	O bone
**78 wt**	F bone	F bone	M2/Cg1	TH_Rt?	TH_LD?	CA1p	hf	DGh/CA3c	CA2o	CA2p	RS?	RS?	O bone
**82 tg**	F bone	F bone	Cg1?	lat ventr	fimbria	CA1r	DGm ub	DGg tip	CA2o	CA2p	RS	RS	O bone
**83 wt**	F bone	F bone	Cg2	TH_Rt	TH_VP	CA1r	DGg ub?	DGm lb	CA1p	CA2r	RS	RS	O bone
**87 wt**	F bone	F bone	M2/Cg1	TH_Rt	TH_VP	hf?	DGg ub?	DGm lb	CA1o	CA1r	RS	RS	O bone
**96 tg**	F bone	F bone	Cg1?	TH_LP	TH_Po	CA1lm	DGg ub	DGg lb	DGm ub	CA3c lu	RS	RS	O bone
**97 tg**	F bone	F bone	Cg1?	TH_Rt	TH_VP	hf?	DGm ub	DGg lb	CA1p	CA1r	RS	RS	O bone
**103 tg**	F bone	F bone	Cg1?	TH_Rt	TH_VPL	DGm up	DGh	DGm lb	CA1p	CA1lm	V2	V2	O bone
**108 wt**	F bone	F bone	M2/Cg1	TH_VP	TH_VP	CA1p	CA1r	CA1r	CA1o	CA1p	RS	RS	O bone
**124 tg**	F bone	F bone	Cg2	TH_Rt	TH_VP	CA1p	hf?	DGg ub?	CA1p	CA1r	RS	RS	O bone

*The top row indicate the channel numbers and the 2^nd^ from the top row the intended locations of the electrodes. FC, frontal cortex; ACC, anterior cingulate cortex; TH, thalamus; RS, retrosplenial cortex; OC, occipital cortex; scr, screw; s, superficial; m, medial; d, deep. Cg1, cingulate cortex; field1; M2, motor cortex; field 2; LD, laterodorsal nucleus; Rt, reticulate n.; LP, lateroposterior n.; VP, ventral posterior n.; Po, posterior n.; VPL, ventral posterior lateral n.; lat ventr, lateral ventricle; CA1o, cornu ammonis; stratum oriens; CA1p, str. pyramidale; CA1r, str. radiatum; CA1lm, str. lacunosum-moleculare; CA3c, CA3 subfield c; DGm, dentate gyrus; str. moleculare; DGg, str. granulosum; DGh, hilus; ub, upper blade; lb, lower blade; hf, hippocampus fissure; CA3lu, CA3 str. lucidum; cc, corpus callosum; V2, visual cortex, field 2*.

### Statistical Analysis

Statistical comparisons were performed in IBM SPSS 21.0. Normality of the data was assessed with Kolmogorov-Smirnov test. If violation of normality was caused by a single outlier, the outlier was removed and normality tested again.

The occurrence of various spike types during movement, immobility and sleep was evaluated by first counting the % of each spike type during each state of the total spike count in each animal. Then these relative counts for movement-related spikes were adjusted for the % of the total time spent in each behavioral state. For instance, if a mouse spent 24% of total recording time moving, the relative spike count during movement (%) was divided by 24. Thus, if 24% of the spikes occurred during movement, this ratio becomes 1, meaning that it matches the expected value. Finally, the distribution of these ratios for each spike type across all animals were compared to 1 using one-sample *t*-test.

When comparing the occurrence of each spike type between the genotypes, the spike count was expressed as the number of spikes per 1 h of immobility. Since the counts varied greatly within each genotype, none of the distributions was Gaussian, and the comparison was done with non-parametric Mann–Whitney test.

The response of various spike types to anti-epileptic drugs was assesses according to within-subject design (three treatments) using ANOVA for repeated measures. Because of large individual variability in the spike counts, a two-way mixed ANOVA model could not be used. To assess potential genotype differences in drug responses, we ran one-way ANOVA for repeated measures either using all animals, or by analyzing the genotypes separately. Only mice with at least one spike of type in question under saline were included in the analysis. For normally distributed data, the *post-hoc* comparison of each of the two active drugs to saline was done with paired *t*-test, otherwise Wilcoxon signed ranks test was used.

Source localization studies were explorative/ mechanistic and did not include group comparisons.

All data were reported as mean ± SEM and the significant level was set at *p* < 0.05.

## Results

### Heterogeneity of Cortical Spike Discharges

**Figure 2:**
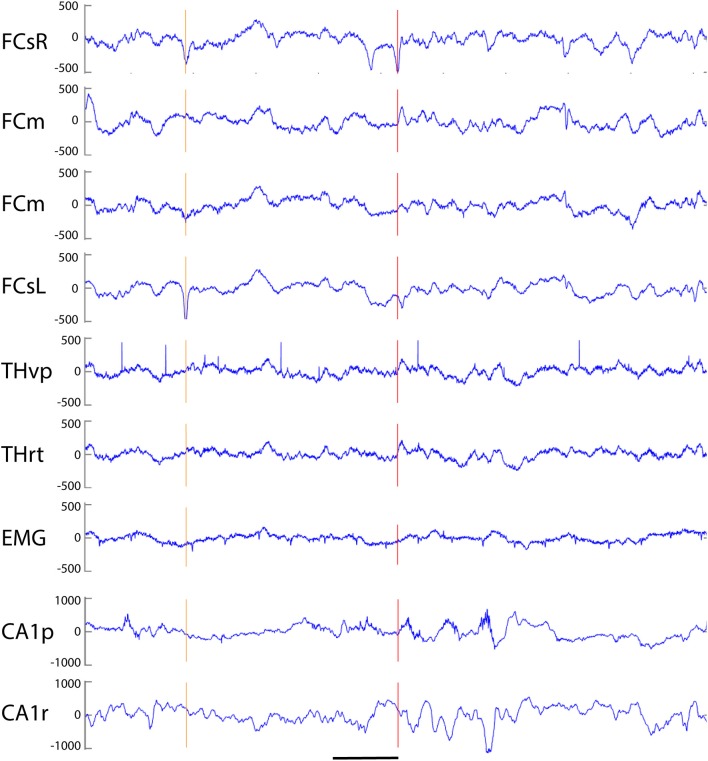
Example of three simple cortical spikes recorded from an APP/PS1 mouse. The top channel (right frontal screw, FCsR) was used as a reference in all spike analyses. Only the 3^rd^ spike exceeded the threshold of 6 SD, and is marked with a red vertical line to help comparison of spike timing between channels. The first one from left (orange vertical line) reached the 6 SD threshold on the left frontal screw electrode (FCsL) and appeared simultaneously but smaller in amplitude on the right frontal screw channel. The second spike is visible on the right (preceding the red vertical line) hemisphere but not at all on the left. None of the spikes is linked with hippocampal spikes. FCsR, right frontal screw; FCm, medial frontal; FCsL, left frontal screw; THvp, thalamus; ventral posterior nucleus; THrt, thalamus; reticular nucleus; EMG, electromyogram; CA1p, CA1 of hippocampus; stratum pyramidale; CA1r, CA1; stratum radiatum. Scale bar, 200 ms.

**Figure 3:**
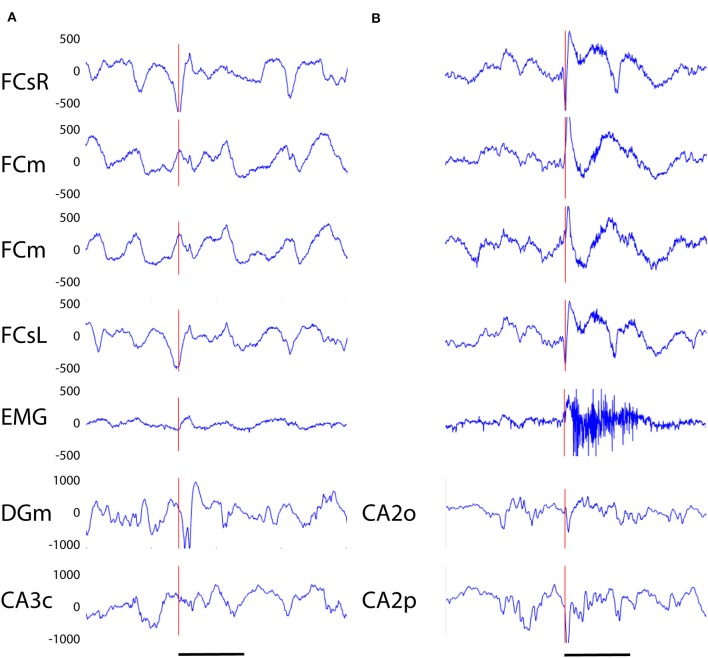
**(A)** Example of a cortico-hippocampal (CH) spike recorded from an APP/PS1 mouse. The spike (vertical red line) begins 2–3 ms earlier on the left frontal screw channel than the right one and is followed by a double spike in the hippocampus (DGm) ~10 ms later. **(B)** Example of a CH spike with a muscle twitch from an APP/PS1 mouse. A simultaneous spike on left and right frontal screw channels is immediately followed by a surface-positive spike on medial frontal channels and a spike on hippocampal channels, and with a slight delay by a 200 ms high-frequency activation of the EMG. Channel abbreviation as in Figure 2, except DGm, dentate gyrus, molecular layer; CA3c, hippocampal pyramidal cell layer CA3c. CA2o, hippocampal CA2, stratum oriens, CA2p, CA2, stratum pyramidale. Scale bar, 200 ms.

**Figure 4:**
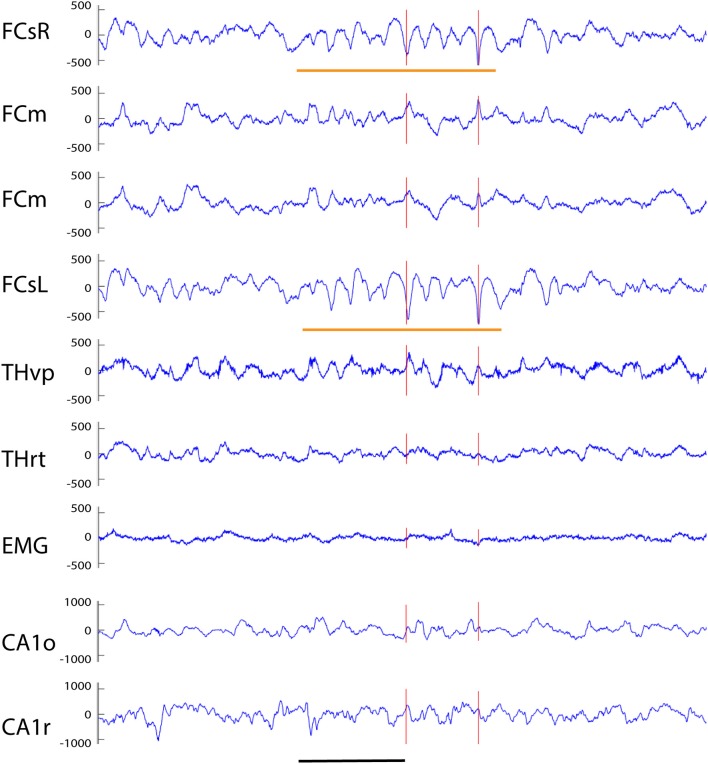
Example of a spindle-associated spikes recorded from a wild-type mouse. A clear sinusoidal spindle oscillation, in this case 11 Hz, can been seen on both frontal screw channels with a 10–15 ms offset (orange horizontal lines). The red vertical lines indicates those spikes on the FCsR reference channel that exceeded the 6 SD threshold. While the subcortical channels do not reflect the oscillation itself the prominent spikes can be seen in reverse polarity on both medial frontal and thalamic THvp channels. Channel abbreviations as in Figure 2. Scale bar, 500 ms.

**Figure 5:**
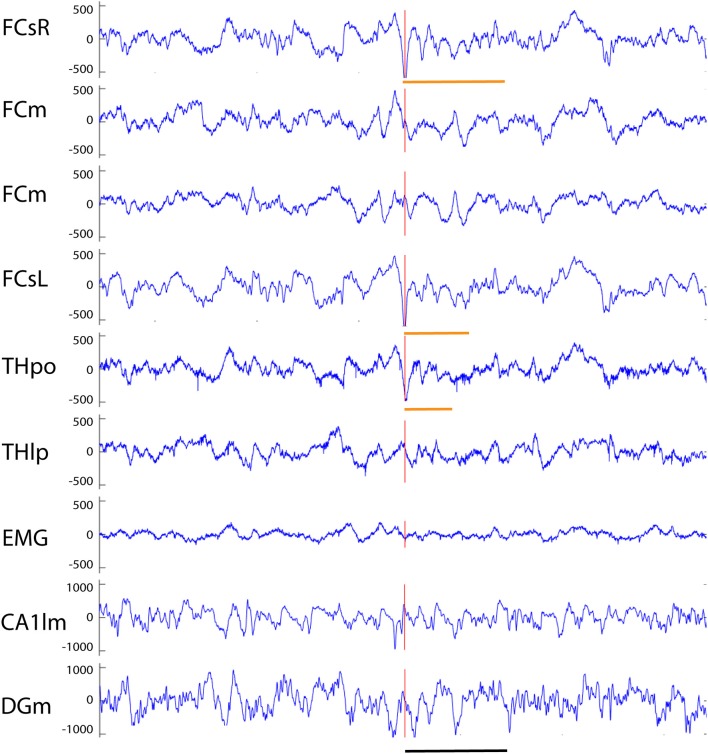
Example of a fast-spindle associated spike recorded from an APP/PS1 mouse. Both frontal screw channels and the thalamic THpo channel show a prominent surface-negative spike (red vertical line) that is followed by a 14 Hz spindle oscillation gradually fading off in amplitude (orange horizontal lines). On FCsR channels 7 cycles of oscillation can be identified, but only 3–4 on FCsL and THpo channels. Channel abbreviations as in Figure 2, except THpo, thalamus, posterior oralis nucleus; CA1lm, CA1 stratum lacunosum moleculare; DGm, dentate gyrus, stratum moleculare. Scale bar = 500 ms.

**Figure 6:**
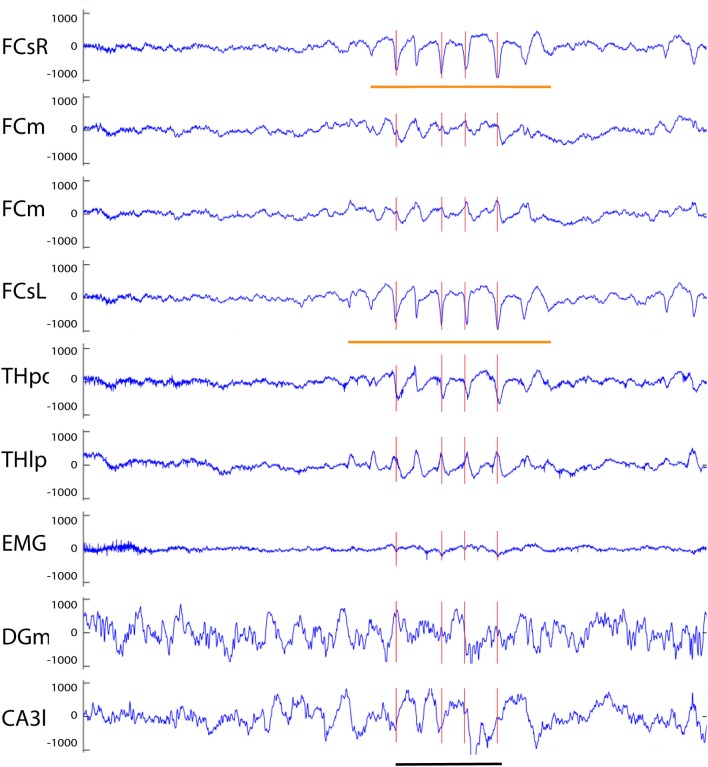
Example of a spike-wave discharge recorded from an APP/PS1 mouse. A prominent complex with surface-negative spikes and surface-positive waves at 8 Hz can been seen in full synchrony on both frontal screw channels (orange horizontal lines). Oscillations of opposite polarity can be seen on both medial frontal channels, and spike of same and reverse polarity on thalamic THpo and THlp channels, respectively, all in full synchrony with the spikes recorded on the cortical surface. The red vertical lines indicate spikes exceeding the 6 SD threshold on the FCsR reference channel. Channel abbreviations as above, except CA3l, CA3 stratum lucidum. Scale bar = 500 ms.

**Figure 7:**
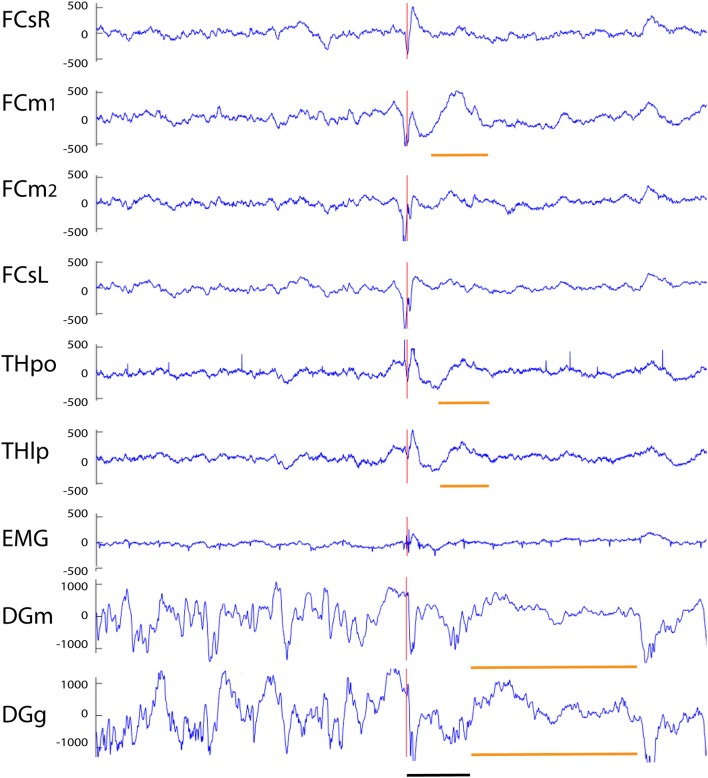
Example of a cortico-hippocampal spike with after hyperpolarization recorded from an APP/PS1 mouse. A spike on FCsL and both FCm channels is followed by a spike on FCsR reference channel 2 ms later (red vertical line). About 10 ms later a spike can be seen on both hippocampal channels (which shows up as a surface-positive deflection in all other channels). This is followed by a slow surface-positive wave lasting for ~ 150 ms on medial frontal and thalamic channels (short horizontal orange line) and up to 600 ms on hippocampal channels (long horizontal orange line). Channel abbreviations as above, except DGg, dentage gyrus, stratum granulare. Scale bar = 200 ms.

**Figure 8:**
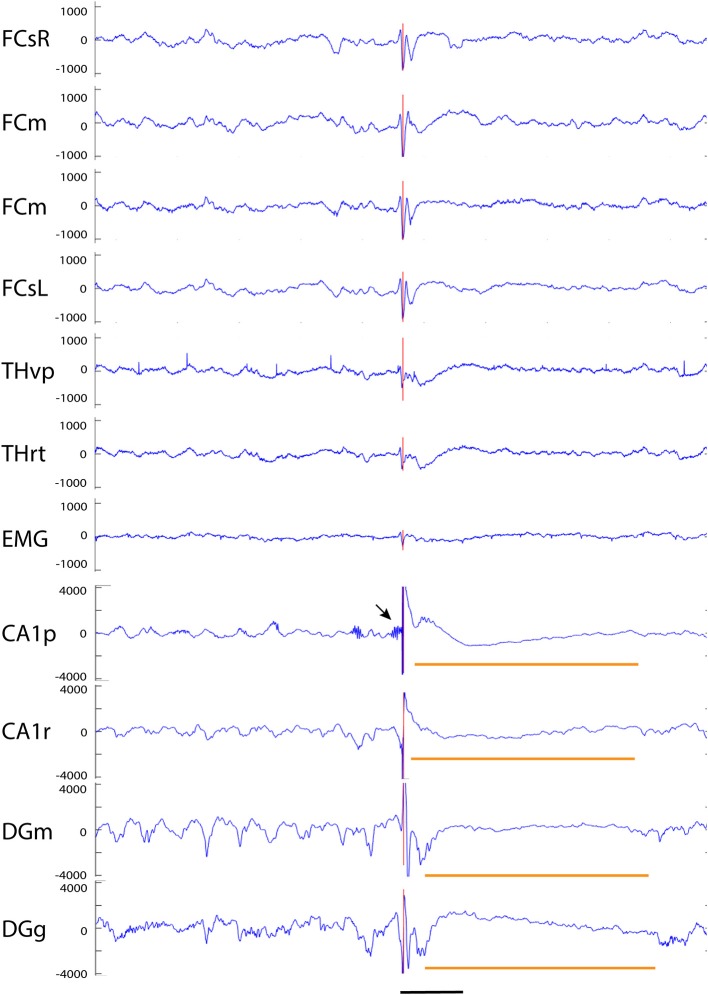
Example of a giant spike recorded from an APP/PS1 mouse. A simultaneous large spike (note a different y-axis scale from previous examples) of varying polarity can be seen on all channels. In addition, there are two surface-positive deflections and a surface-negative one on the spike complex as seen on the cortical screw channels. The negative spike exceeding the 6 SD threshold on the FCsR reference channels is shown with the red vertical line. The fast spikes are followed by a slow negative-positive shift >200 ms on all frontal and thalamic channels (short horizontal orange line). On the hippocampal channels, the most prominent spike reaches an amplitude of > ± 4 mV and is followed by a hyperpolarization response up to 800 ms (long horizontal orange line). In this case, the largest spike arising from the CA1 pyramidal cell layer was preceded by a prominent burst of gamma-activity (arrow) just before the spike onset. Channel abbreviations as before, scale bar = 200 ms.

Our analysis focused on single high-voltage (6 SD or more above baseline) sharp (duration <50 ms) surface-negative spikes that could be detected on the cortical screw electrodes. Surface-positive deviations helped to recognize subcortical activity associated with surface-negative spikes but were not counted *per se*. The single spikes were further classified based on their voltage distribution on the cortical, hippocampal and EMG channels. In addition, we treated as separate category spikes associated with known spike-wave discharges (22), sleep spindles (21), or fast spindles, a novel type of spindle oscillation that we detected in this data set (see Methods for detailed criteria). All surface-negative spikes detected on the cortical screw electrodes were included in one of the following categories. (1) The *cortical spikes* displayed a peak above the threshold (negative) only on the cortical channel. The spikes could be strictly limited to one cortical hemisphere or be bilateral (Figure 2). In the latter case, they may occur simultaneously (within the limits of the resolution) or show a phase-lag between hemispheres (Figures 3, 7). The cortical spikes were often reflected in the thalamic channels, but the thalamic LFPs were highly variable between animals probably due to variable precise location of the thalamic electrodes (see Table 1). Therefore, we could not reliably further separate pure cortical vs. cortico-thalamic spikes in this study. (2) *Cortico-hippocampal (CH) spikes* had a hippocampal spike ± 100 ms before or after the cortical spike (Figure 3A). Cortical or cortico-hippocampal spikes followed by sustained EMG complex 20–100 ms after the peak were defined as (3) *cortical spikes with a muscle twitch* (Figure 3B). Such a spike most likely derives from motor cortex activation and may correspond to clinically defined myoclonus. All these spikes occurred without any particular oscillation pattern in the background. In contrast, (4) *spindle-associated spikes* initiated or were riding on the through of a 10–12 Hz sleep spindle (Figure 4). A related, but distinct pattern constituted of (5) *fast-spindle associated spikes*. Here the spike was present only as the beginning of a 12–14 Hz shorter oscillation that soon faded out (Figure 5). Although both types of spindle oscillations were partially non-overlapping between the hemispheres, the dominant spikes usually occurred bilaterally at the same time. Whereas spindle- associated spikes were single spikes skipping some oscillatory cycles, (6) *spike-wave discharges* (SWDs) were large spikes occurring at every cycle of the underlying 8–11 Hz oscillation (wave). Typically, the complex was asymmetric, such that the surface-negative spikes were much larger in amplitude than the surface-positive waves (Figure 6). These were mostly bilateral and fully synchronized between the hemispheres. Electrodes in certain thalamic nuclei often showed a mirror pattern of opposite polarity (Figure 6). The last two categories of spikes were characterized by “after hyperpolarization,” a sustained shift in the baseline and suppression of high-frequency activity for at least 200 ms after the spike onset, most prominently in the hippocampal channels. These correspond to what has been described as interictal spikes in the literature. Since we could not detect a seizure during the short video-EEG session in any but one of the study mice, we are hesitant to call these spike interictal. Further, these fell into two subtypes (which may represent two ends of the continuum, though). (7) *Cortico-hippocampal spikes with after hyperpolarization* differed from type 2 spikes only in that the spike itself was followed by a slow baseline shift and suppression of fast activity, which in the hippocampal channels could last over 500 ms (Figure 7). (8) *Giant spikes*, as their name indicates, could have massive hippocampus spikes reaching an amplitude of ± 5 mV or 30 SD from the baseline. These typically consisted of a chain of spikes within a short time window spanning from the cortical surface to hippocampus, which resulted in a twisted appearance of the waveform when recorded from the cortical screw electrode. Further, the most prominent spikes seemed to occur simultaneously in all recorded channels (Figure 8). We long considered these spikes caused by muscle twitches, but they do not necessarily associate with any EMG activity, and furthermore, in one control experiment we put the mouse during the recording session in a light plastic cage placed on mechanical piezo sensors below each corner. These did not show any vibrations during the giant spikes.

### Giant Spike Generation in the Hippocampus

**Figure 9:**
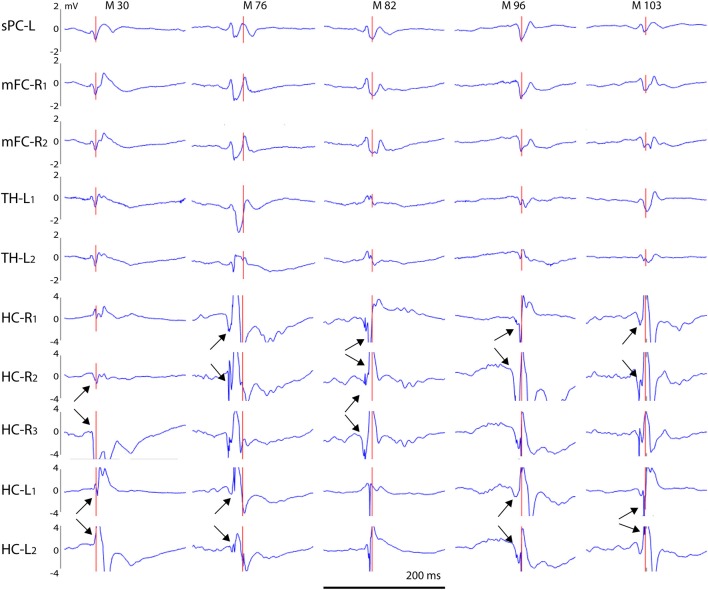
Voltage distribution of giant spikes. A typical giant spike is shown for five individual APP/PS1 mice (M 30–M 103) as recorded on cortical, thalamic and hippocampal channels. Channel abbreviations: sPC-L, left parietal screw; mFC-R, right medial frontal cortex; TH-L, left thalamus; HC-R, HC-L, right/left hippocampus. The numbers 1–3 refer to separate tips of an electrode bundle, with 1 being the most superficial and 3 the deepest. Note that the voltage scale is ± 4 mV for hippocampal channels and ± 2 mV for other channels to compensate the larger extracellular currents in the hippocampus due to its highly layered structure. Arrow pairs point to reversal of the local field potentials indicating a local generator. Scale bar = 200 ms.

**Figure 10:**
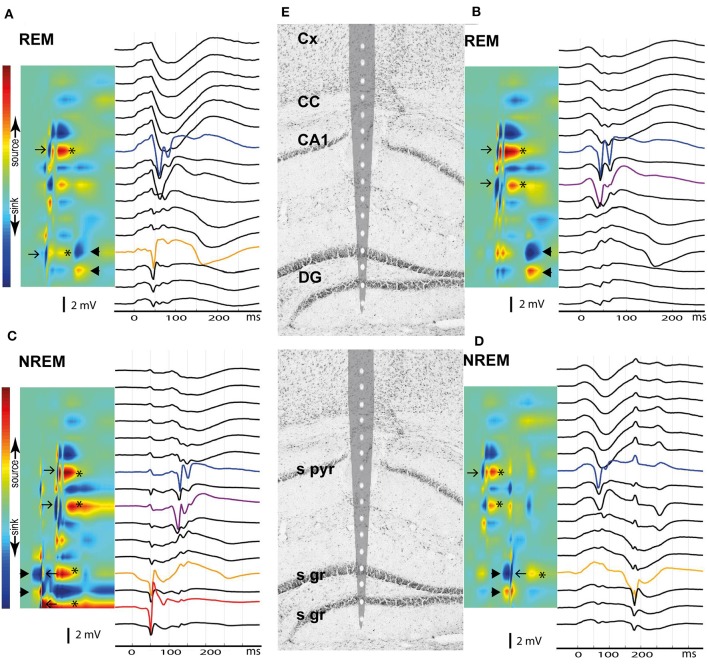
Current source density analysis of the giant spike generators in the hippocampus. Current and voltage distribution across hippocampal layers is shown for four giant spikes **(A–D)** as examples, all aligned with the tip of a 16-channel linear probe repositioned on the hippocampus brain section based on electrolytic marks **(E)**. The top row shows two giant spikes during REM sleep and the bottom row two giant spikes during NREM. **(A)** A large negative population spike in CA1 pyramidal cell layer (CA1p, blue) follows a negative population spike in DG upper granule cell layer (DGg, orange) by ~10 ms. These give rise to brief sinks in the corresponding layers in the CSD map (arrows). Note the spike doublet in CA1p, with attenuation of the second spike. The CA1p and DGg spikes are followed by a sustained hyperpolarization, which results in the strong source in CA1p and DGg channels (*). Finally, ~100 ms after the CA1p spike one sees a sink in the DGg layer extending toward DGm layer and a corresponding source in the DG hilus (arrowheads). This pattern corresponds to perforant pathway (pp) input from the entorhinal cortex. **(B)** Similar population spike doublet in CA1p (blue) as in **(A)** but this time preceded by a population spike in the CA1 stratum radiatum (CA1r, purple), probably due to a dendritic spike. DGg does not show a spike this time, but again ~100 ms after the CA1p population spike, there is a negative wave in the voltage plot and a strong sink in the DGg channel, compatible with pp input. **(C)** This time the giant spike sequence begins with a strong sink in DGg with a source in DG hilus (arrowheads), i.e. pp input. Riding on this wave of depolarization there is a large negative population spike in DGg of the upper blade (orange) and of the lower blade (red), which yield brief sink-source pairs (left-pointing arrows). Immediately after the brief sink in DGg, there is a brief source – sink event, and finally a sustained source in both DGg layers (*). About 70 ms after the DGg spike, there is a negative population spike doublet in CA1r (purple) and in CA1p (blue), which correspond to sinks in the same channels in the CSD plot (right-pointing arrows). These are followed by hyperpolarization (source in CA1p and CA1r, *). **(D)** This giant spike begins clearly in CA1p, giving rise to a brief sink (right-pointing arrow) and a subsequent afterhyperpolarization (source in CA1p and CA1r, *). About 100 ms later, there is a strong sink in DGg and DGm with a source in the DG hilus, corresponding to pp input (arrowheads). Riding on this negative wave, there is a large negative spike in DGg, giving rise to brief sink (left-pointing arrows). The complex ends with a sustained hyperpolarization in upper DGg layer (source, *). Channel abbreviations as before.

No published study so far has been able to identify any epileptic focus in the APP transgenic mice, which makes it difficult to understand the underlying pathophysiology at the molecular level. The giant spikes appeared to have a similar waveform and voltage distribution across channels in an individual mice, and overall, the maximum voltage in the hippocampus in all mice. Furthermore, the earliest local potential reversals were found on hippocampal channels, indicating that the hippocampus is the most probable source of the giant spikes (Figure 9). To further determine the generation of giant spikes, we implanted two APP/PS1 mice with 16-channel linear probes spanning the entire hippocampus, recorded the data at a high resolution (30 kHz), and did a current source density (CSD) analysis to chart their origin. All giant spikes in APP/PS1 mice had a negative population spike in the CA1 pyramidal cell layer (CA1p), often a doublet, so that the second one had a smaller amplitude (Figure 10). These show up in the CSD as brief sinks in CA1p (arrows in Figure 10), followed by a strong and prolonged source right after the second population spike (^*^ in Figure 10), corresponding to an after hyperpolarization. A similar pattern has been demonstrated during interictal spiking in the rat hippocampus after fimbria-fornix transection. In this rat model, there is synchronous firing of multiple pyramidal cells during the CA1p population spike and sustained firing of interneurons during the after hyperpolarization (26). This after hyperpolarization likely accounts for the attenuation of the second CA1p population spike and prevents further spiking during the next ~100 ms. These brief sinks in CA1p were accompanied by somewhat wider negative population spikes in CA1 stratum radiatum (CA1r, arrows in Figure 10), probably corresponding to dendritic spikes of the CA1 pyramidal cells. Most giant spikes also included a negative population spike in the granule cell layer (mainly of the upper blade). The DG spike never occurred during the maximum spike in CA1p but coincided with a small source-sink-source complex, with sources in CA1p and CA1r (Figures 10A,C,D). These likely represent entorhinal input to both DG and CA layers. The main DG population spike could equally well precede or follow the CA1p spike. During NREM sleep, the DG population spike occurred as part of a strong sink in the upper DG granule cell and molecular layer, corresponding to perforant path (pp) input (arrowheads in Figure 10). Notably, independent of the sleep state, the CA1p spike was also followed by a similar strong sink in the upper DG granule cell and molecular layer (arrowheads in Figure 10). Since a full cycle of the trisynaptic pathway through the hippocampus – entorhinal cortex circuitry is estimated to take only ~25 ms (26), the coupling of CA1p population spike and pp input to DG molecular layer cannot be explained by direct CA1 input to entorhinal cortex. In fact, the interval between the two CA1p spikes was ~25 ms and can reflect oscillation in the trisynaptic pathway. Most likely the delay between CA1p spike and pp input results from the prolonged after hyperpolarization. Taken together, the CSD analysis shows that a giant spike is composed of series of depolarization—hyperpolarization events at different nodes in the entorhinal—hippocampal circuitry, which also explains the complex shape of the extracellular field.

### Most Spike Discharges Occur During Immobility

Based on the video analysis we could determine the movement of the center of mass of the mouse body at each moment of the recording. Accordingly, we determined the occurrence of various spike types during three behavioral states, movement, waking immobility and sleep. The mice had experienced several familiarization sessions in the recording environment before the actual recordings took place, so it was natural that they spent most of the time (88% on average) immobile and 57% of the time on average in sleep. Nevertheless, there was a huge variation between individual animals and between days, so that the time spent in sleep varied between 8 and 88% between recording sessions. To assess the behavioral state of the mice during the spike occurrence, we first calculated the % of spikes occurring during in each behavioral state in each recording session (only sessions with saline injections). Then we calculated odds ratios for the spikes to occur during a particular state in each mouse by dividing the % spikes in each state by the % time spend in that state. Table 2 summarizes the findings for each spike type. In fact, all spike types occurred far less frequently during movement than immobility (*p* < 0.001, one-sample *t*-test). Fast-spindle associated spikes, cortico-hippocampal spikes with after hyperpolarization and giant spikes never occurred during movement. Further, all spike types except SWDs occurred less frequently during waking immobility than sleep (*p* < 0.001, one-sample *t*-test).

**Table 2A:** Occurrence of various spike types in the three behavioral states: sleep, waking immobility, and movement.

**Type**	**C %**	**CH %**	**C+M %**	**SPL %**	**FSPL %**	**SWD %**	**CH+hp %**	**GS %**
Sleep	83.0 ± 3.9	91.4 ± 2.6	79.5 ± 4.8	93.4 ± 3.9	91.8 ± 5.1	68.0 ± 7.6	99.6 ± 0.4	87.3 ± 8.0
Immob	16.6 ± 3.8	10.2 ± 2.7	14.2 ± 3.5	7.0 ± 4.2	8.2 ± 5.1	31.1 ± 7.4	0.4 ± 0.4	8.2 ± 5.1
Move	0.4 ± 0.3	0.3 ± 0.2	4.2 ± 4.2	0.1 ± 0.0	0.0 ± 0.0	0.9 ± 0.5	0.0 ± 0.0	0.0 ± 0.0

*The figures are mean ± SEM% of all spikes recorded during saline sessions. C, cortical sp; CH, cortico-hippocampal sp; C+M, cortical sp + movement; SPL, spindle associated sp; FSPL, fast spindle associated sp; SWD, spike-wave discharge; CH+hp, cortico-hippocampal sp with afterhyperpolarization; GS, giant sp*.

**Table 2B:** Odds ratios of spikes occurring in the three behavioral states during saline sessions.

**Type**	**C**	**CH**	**C+M**	**SPL**	**FSPL**	**SWD**	**CH+hp**	**GS**
Sleep	1.56 ± 0.09^***^	1.67 ± 0.13^***^	1.42 ± 0.15^*^	2.04 ± 0.43^*^	1.66 ± 0.21^**^	1.23 ± 0.19	1.67 ± 0.14^***^	1.56 ± 0.13^***^
Immob	0.48 ± 0.08^***^	0.25 ± 0.07^***^	0.52 ± 0.09^***^	0.14 ± 0.07^***^	0.20 ± 0.12^***^	1.00 ± 0.23	0.02 ± 0.02^***^	0.03 ± 0.02^***^
Move	0.01 ± 0.01^***^	0.02 ± 0.01^***^	0.10 ± 0.10^***^	0.01 ± 0.00^***^	0.00 ± 0.00^***^	0.03 ± 0.01^***^	0.00 ± 0.00^***^	0.00 ± 0.00^***^

*The proportion of spikes per each behavioral state is divided by the time the mice spent in the corresponding state. Means ± SEMs are shown*.

* *p < 0.05*,

** *p < 0.01*,

*** *p < 0.001 (one-sample t-test against odds ratio 1)*.

### Giant Spikes Occur During Both REM and NREM Sleep

To more closely assess the behavioral state during the occurrence of giant spikes, we went through the video recordings and LFP traces around all identified 160 giant spikes in our material. In fact, all but one giant spike were recorded during sleep states (NREM 87, REM 67, transition state 5). Although more giant spikes were detected during NREM sleep than during REM sleep, considering the shorter duration of REM epochs, the likelihood of detecting a giant spike was practically equal between NREM and REM. In addition, we analyzed separately those giant spikes that appeared in a cluster of 2 to 5 spikes, so that the maximum inter-event time was 5 s. Four APP/PS1 mice showed such clusters comprising altogether 35 giant spikes. Interestingly, those were predominantly present during REM sleep (26 out of 35; chi^2^ = 10.8, *p* = 0.001 for the NREM vs. REM comparison).

### Only SWDs, CH-Spikes With After Hyperpolarization and Giant Spikes Were Clearly Overrepresented in APP/PS1 Mice

Having identified different spike categories, we went on to test whether they are differentially associated with the epileptic APP/PS1 genotype. To this end, we pooled video-EEG recordings on two consecutive days (3 h per day) with saline injections. Since spikes were more common during immobility while the level of motor activity varied between animals and between days, we normalized the spikes counts to the immobility time and expressed them as number of spikes per 1 h of immobility.

**Figure 11:**
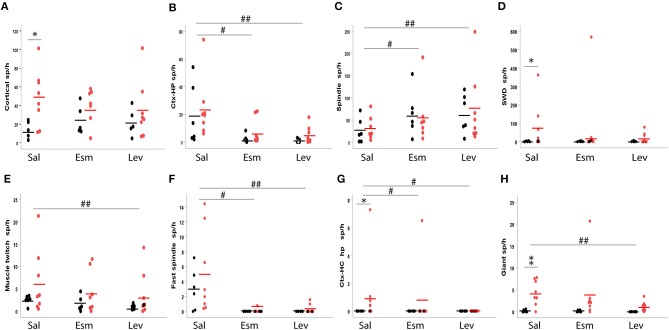
Occurrence of various spike types in wild-type (black) and APP/PS1 (red) mice. The number of spikes is given per 1 h of immobility, since most spikes occurred during immobility (sleep). The top row depicts frequent spike types and the bottom row less frequent ones. **(A)** Cortical spindles, **(B)** cortico-hippocampal spikes, **(C)** spindle-associated spikes, **(D)** spike-wave discharge related spikes, **(E)** spike with muscle twitches, **(F)** fast spindle associated spikes, **(G)** cortico-hippocampal spikes with afterhyperpolarization, **(H)** giant spikes. Sal, saline; Esm, ethosuximide; Lev, levetiracetam. *Significant different between the genotypes, *p* < 0.05, **p < 0.01. ^#^Significant treatment effect, *p* < 0.05, ^##^*p* < 0.01.

One wild-type mouse displayed single unilateral cortical spikes roughly once every 20 s during both movement and immobility throughout the 3-h recording session and was removed from the analysis of cortical spikes as an outlier. Otherwise, all mice and all recording sessions were included. Nevertheless, the counts of spikes of each type were highly heterogeneous between individual mice, but consistent between the two saline sessions for an individual mouse (see Supplemental Table 2 for details). Therefore, the genotype comparison had to be done with non-parametric testing. The occurrence of cortical spikes with muscle twitches, CH-spikes and both spindle-associated spikes did not differ between the genotypes (Figure 11). Cortical spikes were slightly more common in APP/PS1 mice (*p* = 0.045), while SWDs (*p* = 0.04), CH spikes with after hyperpolarization (*p* = 0.02) and giant spikes (*p* = 0.008) were detected almost exclusively in APP/PS1 mice, but only in a subset of them as seen from large error bars in Figure 11.

### The Spike Types Differ in Their Response to Ethosuximide and Levetiraceram

Next, we tested how various spike discharge subtypes respond to anti-epileptic drugs (AEDs). For this purpose, we chose levetiracetam (LEV), which has proven to be most effective against seizures in APP transgenic mice and a cognitive enhancer at a non-sedative dose (13), and ethosuximide (ESM) as a prototype drug effective against absence seizures. Simple *cortical spikes* proved to be resistant to these treatments [All: *F*_(2, 12)_ = 0.7, *p* = 0.51; WT: *F*_(2, 3)_ = 1.9, *p* = 0.29; TG: *F*_(2, 6)_ = 0.7, *p* = 0.53], but *CH-spikes* did respond to both AEDs [All: *F*_(2, 12)_ = 10.6, *p* = 0.002; WT: *F*_(2, 4)_ = 3.8, *p* = 0.12; TG: *F*_(2, 6)_ = 5.9, *p* = 0.04; Figure 11]. In contrast, *cortical spikes with muscle twitches* responded to AEDs only when the genotypes were pooled and only for LEV in the *post-hoc* test [All: *F*_(2, 12)_ = 7.5, *p* = 0.008; WT: *F*_(2, 4)_ = 4.8, *p* = 0.09; TG: *F*_(2, 6)_ = 3.9, *p* = 0.08; Figure 11]. Interestingly, the number of *spindle-associated spikes* increased in response to both AEDs [All: *F*_(2, 12)_ = 4.5, *p* = 0.035; WT: *F*_(2, 4)_ = 7.5, *p* = 0.04; TG: *F*_(2, 6)_ = 1.5, *p* = 0.30], while *fast-spindle associated spikes* showed an opposite response [All: *F*_(2, 12)_ = 8.9, *p* = 0.035; WT: *F*_(1, 5)_ = 6.9, *p* < 0.05; TG: *F*_(2, 6)_ = 7.2, *p* = 0.003, Figure 11]. *SWDs* did not respond to AED treatment [All: *F*_(2, 6)_ = 2.0, *p* = 0.21; WT: *F*_(2, 4)_ = 0.4, *p* = 0.70; TG: *F*_(2, 6)_ = 2.0, *p* = 0.22]. Only five APP/PS1 mice displayed *CH-spikes with after hyperpolarization*. These were responsive to both ESM and LEV (both *p* < 0.05, Wilcoxon signed-rank test). Notably, these spikes were not present at all during LEV treatment (Figure 11). LEV was also effective in suppressing *giant spikes* in APP/PS1, while ESM had mixed effects [TG: *F*_(2, 6)_ = 8.8, *p* = 0.02; ESM *p* = 0.88, LEV *p* = 0.004; Figure 11]. Due to the high inter-individual variability in the spike occurrence, we could not apply a two-way ANOVA model. In as much as spikes were present in wild-type mice, their response to AEDs seemed to follow the pattern of APP/PS1 mice (Figure 11).

### Presence of Giant Spikes Indicates a High Risk for Seizures

**Figure 12:**
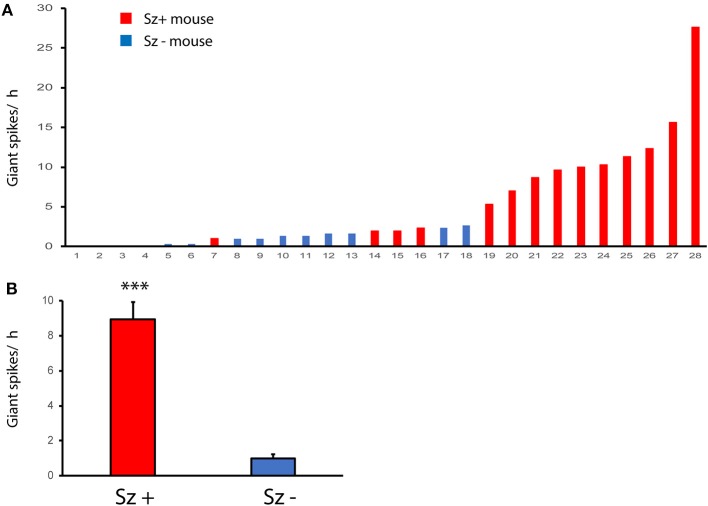
Giant spikes are related to the risk of seizures. **(A)** Number of giant spikes per hour in individual APP/PS1 mice with identified seizures (Sz +, *n* = 14) and seizure free mice (Sz -, *n* = 14) during a 2-week video-EEG recording. The dashed line indicates a threshold above which all mice express seizures. **(B)** The same dataset as in **(A)** but comparison between Sz+ and Sz – groups. ***Significant difference in the number of giant spikes, Mann–Whittney test, *p* < 0.001.

Finally, we wanted to know whether giant spikes are related to seizures and whether they could be used as a surrogate marker for seizures that *per se* are rare and difficult to detect. To this end, we utilized our video-EEG archives from three studies on the effect of antiepileptic treatments in the same APP/PS1 mouse model (12, 24, 25). Having first validated parameters that reliably (sensitivity 92%, specificity of 91%) identify giant spikes on the cortical screw channels only in the present multichannel dataset (see section Materials and Methods), we screened 3 h of EEG preceding a seizure in the archived files of 14 APP/PS1 mice (Sz + mice) for the number of giant spikes. As a comparison, we sought a matched pair (Sz – mice) for each mouse from the same study cohort without any identified seizures during the 2-week baseline measurement and counted the number of giant spikes during the same 3 h time window as for the Sz + mouse. As illustrated in Figure 12B, the number of giant spikes was significantly higher in Sz + mice than in Sz – mice (*p* < 0.001, Mann–Whitney test). Figure 12A plots the number of detected giant spikes during the 3-h follow-up in every single mouse in the study. It appears that mice showing fewer than 10 giant spikes per 3 h could equally well belong to Sz + as Sz – groups. However, every single mouse in this sample showing more than 5 giant spikes/ h was found to have at least one seizure. This sample suggests that the seizure risk increases non-linearly with the frequency of giant spikes.

**Figure 13:**
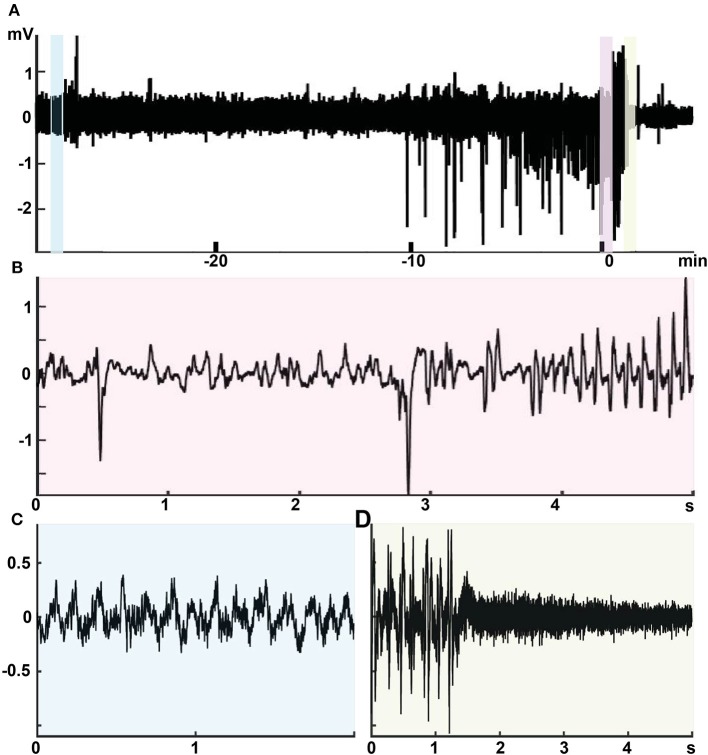
Intimate relationship between giant spikes and a seizure in a 9-month-old male APPswe/PS1dE9 mouse. Recording from one channel of a movable tetrode located in the CA1 pyramidal cell layer. **(A)** An overview of the recording session at a low time resolution. Time 0 is the seizure onset. During the first 40 min up to time −10 min in the figure, the mouse was in a constant movement and the LFP shows regular theta rhythm with overriding gamma oscillation (inset **C**, light blue). Then the mouse fell asleep and the LFP shows frequent giants spikes during ~10 min. The last giant spike is continuous with the onset of a 32-s generalized seizure (inset **B**, pink). The seizure ends with a total suppression of the LFP for ~40 s (inset **D**, light green).

Further evidence for the connection between giant spikes and seizure comes from two rare cases (the only ones during 3 years of multichannel recording in APP/PS1 mice in our laboratory) where the mouse was recorded the day before a sudden death, which is a well-known phenomenon among APP transgenic mice. The first one was a 4.5-month-old APP/PS1 male mouse with cortical and hippocampal electrodes that showed as many as 245 giant spikes during its first 3-h recording session. Next morning, the mouse was found dead in its home cage in an extended posture, and the autopsy did not reveal anything unusual. This is a typical premature sudden death found in colonies of APP transgenic mice and linked with prolonged seizures are a most likely cause (27, 28). Another case was a 9-month-old APP/PS1 male mouse with drivable tetrodes aimed at hippocampal CA1. On the first recording day, the mouse was constantly in movement for 40 min before falling asleep. Then it displayed 62 giant spikes during 10 min of sleep (both NREM and REM) followed by a classic seizure of 32 s in duration with a post-ictal suppression (Figure 13). Next day, it displayed 237 giant spikes during 2 h, being mostly asleep. The following morning, the mouse was found dead in its home cage as the first mouse.

## Discussion

Since spontaneous seizures in APP transgenic mice are rare and their detection requires weeks of continuous video-EEG and large study groups to assess treatment responses, most pharmacological intervention studies have used “epileptic spikes” recorded with epidural cortical electrodes as surrogate markers for the epilepsy phenotype. However, there is no consensus as to which features in the standard epidural cortical recordings make single spikes in EEG “epileptic” in the transgenic AD mice and not even a generally accepted definition for a “spike.” Based on previous publications in APP transgenic mice (9, 13, 14) we adopted relative strict amplitude (>6 SD) and duration (<50 ms) criteria for surface-negative spikes recorded with standard cortical screw electrodes to be included in our study encompassing 15 842 spike discharges. Our recordings with multiple implanted electrodes show that a single large spike recorded with a skull screw electrode in a mouse can equally well be generated locally in the cortex or be part of an avalanche of discharges in a large network.

To our knowledge, this the first attempt to classify spikes detected on skull-EEG in the mouse into subcategories. We first assessed whether a surface-negative spike was detected only locally in the cortical channels (frontal or parietal referred to the occipital bone) or whether it was accompanied by a locally generated spike or sharp wave in the thalamus or hippocampus ±100 ms from the peak time. Since the hippocampus is a uniformly layered structure that generates strong extracellular fields, it was easy to detect locally generated LFPs with a staggered triple electrode and often even with a double electrode. Therefore, we were able to identify several stereotypic patterns of LFPs occurring simultaneously in the cortex and hippocampus. In contrast, we got highly variable recording from the thalamus, which has irregular and mainly oval shaped nuclei and weak extracellular fields. Due to this anatomy, it was difficult to get electrodes in the same nuclei with only stereotactic coordinates. We found some interesting cases of cortical only vs. thalamocortical spikes, but due to the variability between mice, we could not use the thalamic channels as a basis of categorization of individual spikes. Instead, based on a characteristic EMG signal instantly following some cortical spikes, we could identify a stereotypically repeating pattern of a cortical spike (with or without hippocampal involvement) followed by a short EMG increase. Based on the video, this EMG signal was associated with a small head movement. Phenomenologically these made a category of their own but we cannot yet say if the generating circuitry differs from spikes without the EMG activity. Notably, to isolate individual spikes and reliably determine their amplitude in terms of SDs of baseline, we had to filter out large amplitude slow oscillations. Based on systematic exploration, we found that a smooth 2nd order high-pass filtering at 8 Hz did not distort the shape of spikes or spindle oscillations, and only attenuated the theta rhythm. We could thus identify well-known sleep spindles (21) and SWDs (22). Often individual surface-negative spikes were located precisely at the trough of the sleep spindle suggesting a tight relationship between them, while repeating spiking is an inherent part of the SWD structures. Therefore, we separated spikes that were part of these oscillations. In addition, we identified a novel type of fading cortical oscillation that we named a fast-spindle, which was always triggered by a single spike. Based on these additional features, we could identify eight different spike types that differed in terms of their underlying circuitry, specificity to the APP/PS1 genotype and response to common anti-epileptic drugs (AEDs). Simple cortical spikes could be detectable only with the cortical screw electrode on one hemisphere speaking for a local generation, while spindle-associated spikes and spike-wave discharges (SWDs) activated a cortico-thalamic circuitry, and giant spikes involved all recorded brain regions. Cortico-hippocampal (CH) spikes, spikes with muscle twitches and spikes associated with regular or fast spindles could be detected to the same extent in wild-type and APP/PS1 mice, whereas SWDs, CH-spikes with after hyperpolarization and giant spikes were almost exclusively present in APP/PS1 mice, but not in all of them. AEDs reduced the occurrence of many spike types but did not significantly influence simple cortical spikes or SWDs, and even increased the occurrence of spindle-associated spikes. This evidence strongly suggests that a better understanding of the underlying circuitry, the relationship between spiking and amyloid pathology, and testing the effect of AEDs all benefit from classification of cortical spikes into functional categories. This report is not meant to provide a comprehensive categorization of epileptic spikes on epidural recordings, but rather should be seen as the first attempt to refine the spike analysis.

Revealing the spike generators in APP/PS1 mice is an important clue to work out cellular and molecular mechanisms that link the epileptic phenotype of APP transgenic mice to downstream consequences of Aβ deposition in the brain. According to present findings, both the neocortex and hippocampus appear as the critical brain regions in this regard. We have earlier demonstrated a sustained depolarization shift in both cortical pyramidal and dentate granule cells in acute brain slices of APPswe/PS1dE9 mice, which can be mimicked by incubating brain slices of wild-type mice with protofibrillar but not by monomeric Aβ42 peptide (6). Parvalbumin (PV) positive basket cells that impinge on the axonal initial segment of pyramidal or granule cells exert the most powerful inhibition to their firing. Thus, loss of PV cells would have a dramatic impact on neuronal excitability. Although reduction of PV cells has been reported in APPswe,ind (TgCRND8) mice [but only in CA3; (29)], no change in PV neuron number was found in DG of 3-month-old APPswe/PS1dE9 mice, and their number in CA1 and CA3 was even increased (30). On the other hand, decreased levels of interneuron specific (PV-cell predominant) voltage-gated Na^+^-channel subunit Nav1.1 has been found in the parietal cortex and its contribution to hyperexcitability shown in 4–7-month-old APPswe,ind (J20) mice (31). However, it remains to be investigated whether the reduction of Nav1.1 applies to hippocampal PV cells and to other mouse models as well. Alternative, increased excitability could arise from increased glutamate release. Indeed, APPswe/PS1dE9 mice show increased glutamate release in the hippocampus even before amyloid plaque formation when Aβ is still in a soluble (or membrane-bound) form (32). There are probably several underlying mechanisms to this increased release. For instance, it is well-documented that Aβ42 through binding to α7 nicotinic acetylcholine receptor can increase glutamate release (33). Furthermore, a recent paper reported a robust overexpression of hippocampal adenosine A_2A_ receptors in AD patients and APPswe/PS1dE9 mice (34). These receptors are mainly presynaptically located and increase glutamate release. The fact that most spike types were inhibited by levetiracetam, which inhibits presynaptic Ca^2+^ channels thereby reducing glutamate release (35), further speaks for increased glutamate release as a key underlying mechanism for spike generation in APP/PS1 mice. On the other hand, many spike types also responded to ethosuximide that reduces T-type Ca^2+^ currents in thalamic neurons and persistent Na^+^ and Ca^2+^ -activated K^+^ currents in thalamic and layer V cortical pyramidal neurons (36). Thus, it is likely that more than one mechanism accounts for the increased neuronal excitability in APP/PS1 mice leading to spike generation. It also remains open why APP/PS1 mice with relatively uniform amyloid pathology (Supplemental Figure 1) display distinct spike types, such as mouse 124 showing hundreds of SWDs but no giant spikes, while mouse 97 showed tens of giant spikes but only a couple of SWDs (Supplemental Table 1).

We found several spike types in the mouse EEG that have not been described in the literature before. Previous studies addressing frequency of cortical spikes in APP transgenic mice have tried to set the detection threshold high enough to avoid recording sleep spindles. Nevertheless, we could see solitary larger spikes that were entrained with the spindle oscillation. They could initiate the entire 10–12 Hz spindle or appear in the middle or in the end of it. However, we also found a faster spindle (12–14 Hz) with a lower amplitude, usually preceded by a single large spike (here called fast-spindle associated spikes). The distinction between these two seemed justified, since both ESM and LEV increased the occurrence of spindle-associated spikes but decreased (and LEV totally eliminated) the occurrence of fast-spindle associated spikes. However, both types of spindle- associated spikes appear to be a normal feature of C57Bl/6J mouse EEG, since they were not overrepresented in APP/PS1 mice. Since the spindle-associated spikes were the most frequent ones recorded, it is important to exclude them when assessing cortical spikes with relevance to AD-related epilepsy.

Another novel spike type is the giant spike. Large hippocampal spikes (up to ~10 SD above baseline) synchronous with cortical spikes and followed by a ~ 200 ms hyperpolarization wave were recently described in the APPswe (Tg2576) mouse (37). These spikes, called by the authors interictal spikes, closely resemble our CH spikes with after hyperpolarization. The giant spikes differed from these in their large amplitude [10–30 SD above baseline], twisted waveforms caused by several population spikes in sequence and long after hyperpolarization up to 1 s. It is possible that CH spikes with after hyperpolarization and giant spikes constitute a continuum and differ only in the extent of underlying synchrony of involved neurons. Interictal spikes in the Kam et al. study were almost exclusively present during REM sleep while giant spikes in our study were most often present during REM sleep, although also frequently found during NREM sleep as well. Interestingly, the strong sleep association with these spikes corresponds to recent findings that epileptiform discharges in AD patients occur predominantly during sleep, and especially during NREM sleep (38).

Our multielectrode recordings suggest that giant spikes in APP/PS1 mice were generated in the hippocampus, which is known to be the brain site with lowest epileptogenic threshold (39). First, in the recordings with distributed electrodes, the largest population spikes and after hyperpolarization were always found in the hippocampus. Second, recordings with triple or double electrodes in the hippocampus revealed clear spike reversals in the hippocampus in most giant spikes (Figure 9). Third, recordings with linear silicone probes revealed a consistent population spike in the CA1 pyramidal cell layer, usually accompanied by another population spike in the DG granule cell layer (CA3 not recorded). The finding of several local sink-source pairs in various hippocampal layers speak for the idea of sequential activation during the giant spike (26, 40). However, the timing between the DG and CA1 population spikes was random, speaking for the idea of independent entorhinal—DG and CA3-CA1 circuitries underlying the giant spikes (40). The presence of several activation loops explains the signature of the giant spike, the twisted waveform in the epidural recordings.

The relationship between interictal spikes and seizures has been debated for decades (41). They have been considered indices of brain hyperexcitability and predictors of seizures, while the other view is that they are protective against seizures as sometimes decrease in interictal spiking precedes the seizure onset (41). Our data in APP/PS1 mice support the idea that high number of interictal spikes increases the risk of seizures. First, our video-EEG archive in APP/PS1 mice revealed than a low frequency of occurrence (in this sample <5 per h) could be present without detected seizures, but that all mice exceeding the threshold also exhibited seizures in long-term video-EEG. Second, we presented two rare cases with hippocampal recordings the day before an APP/PS1 mouse was found dead probably due to a prolonged seizure. Both exhibited a huge number of giant spikes during sleep epochs, the other one just before a verified seizure. Although these are only individual and probably extreme cases, they prove that a very high number of giant spikes can precede a seizure. It is possible that the relationship between giant spikes and seizures is indirect and only reflects modulation by a common underlying factor as suggested by Karoly et al. (41). Giant spikes can be compared to extrasystolic complexes in the electrocardiograph associated with premature ventricular contractions. Occasional extrasystolic complexes are benign but their frequent occurrence may be a manifestation of an underlying heart defect that may lead to fatal ventricular fibrillation.

From the AD viewpoint, the giant spikes may be translationally much more relevant than seizures in AD mouse models. First, convulsive seizures are rare also in AD patients and not considered a major clinical problem. In contrast, interictal spikes can occur up to 20 times/h (3). Interestingly, they occur mainly during stage 2 or 3 NREM sleep (3, 38), which is considered the most important sleep phase for memory consolidation at the systems level, i.e., for transfer of newly learned material from temporary stores in the hippocampus to long-term storage in the cortical networks (42). In a rat kindling model, interictal spikes that occur during a sleep epoch between the learning and test phase of a memory task can impair memory consolidation by inducing false coupling between the hippocampus and cortex (43). Similarly, interictal spiking in AD patients may contribute to forgetting of recent events. Moreover, the only published follow-up study on epileptiform discharges in AD patients so far found significantly faster cognitive decline over 5 years in patients with recorded epileptiform activity than in those without (3). Although the link between epileptiform discharges and cognitive decline in this study was indirect, this report suggests that detected epileptiform activity (usually spikes) in MCI/AD patients by itself should be considered an indication for treatment. In search for optimal pharmacological treatment for non-convulsive epileptiform activity, the giant spikes in APP/PS1 appear a useful and valid outcome measure. The giant spikes arising from the hippocampus closely resemble recently published interictal spikes in AD patients recorded with foramen ovale electrodes (44). As in patients, they occur almost exclusively during deep sleep. They are very rare in wild-type mice but have been so far documented in two common transgenic mice displaying amyloid plaques, the present APPswe/PS1dE9 mice and the APPswe (Tg2576) mouse in a previous report (37). Since giant/interictal spikes are much more specific for AD-like brain amyloidosis that cortical spikes in general, we recommend future preclinical studies in AD model mice to focus on them as the primary outcome measure.

## Data Availability Statement

The datasets generated for this study are available on request to the corresponding author.

## Ethics Statement

The animal study was reviewed and approved by Animal Experiment Board of Finland.

## Author Contributions

IG performed all electrode implantations except for drivable tetrodes. IG and II built all EEG/LFP electrodes and performed all video-EEG recordings and the first round of data analysis. AL build and implanted the drivable tetrodes and conducted recordings with them. SZ and NJ did all the movement analysis from video recordings and its synchronization with spike data. SZ and II analyzed giant spikes and seizures in archived recordings performed by SZ. KG wrote all algorithms for the spike analysis. IG and HT did the final analysis, wrote the manuscript, and prepared the figures. HT planned the experiments.

### Conflict of Interest

The authors declare that the research was conducted in the absence of any commercial or financial relationships that could be construed as a potential conflict of interest.

## Supplementary Material

Supplemental Figure 1 Examples of the regional distribution of amyloid plaque pathology in four APPswe/PS1dE9 mice at the age of 6 months. In all individual mice, the highest amyloid load is in the neocortex, followed by the hippocampus and amygdala. In contrast, the thalamus, hypothalamus, and basal ganglia are devoid of plaques at this stage.

Supplemental Table 1 Spikes types by individual mice.
