# Supplementary figures and images for: Characterization of Epileptic Spiking Associated With Brain Amyloidosis in APP/PS1 Mice
Source: Front Neurol. 2019 Nov 12;10:1151. doi: 10.3389/fneur.2019.01151 (PMC6861424; doi:10.3389/fneur.2019.01151)

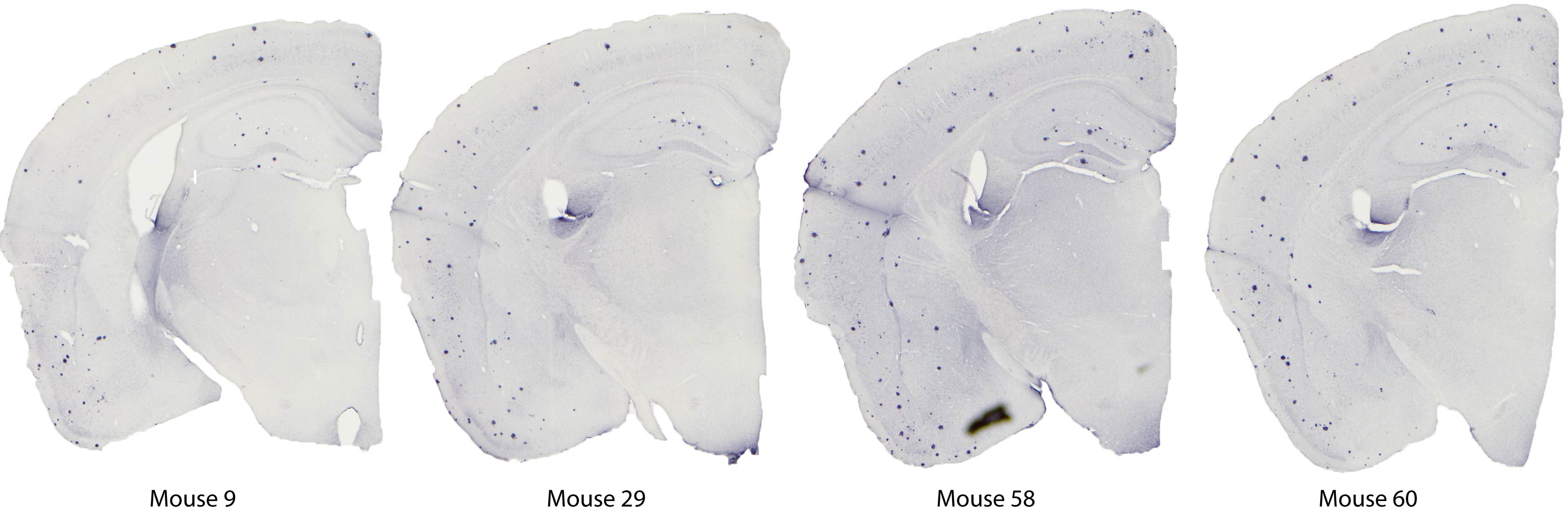

Supplement: Supplemental Figure 1 — Examples of the regional distribution of amyloid plaque pathology in four APPswe/PS1dE9 mice at the age of 6 months. In all individual mice, the highest amyloid load is in the neocortex, followed by the hippocampus and amygdala. In contrast, the thalamus, hypothalamus, and basal ganglia are devoid of plaques at this stage. [file Image_1.JPEG]
