# Supplementary material for: Characterization of Epileptic Spiking Associated With Brain Amyloidosis in APP/PS1 Mice
Source: Front Neurol. 2019 Nov 12;10:1151. doi: 10.3389/fneur.2019.01151 (PMC6861424; doi:10.3389/fneur.2019.01151)
Supplement: Supplemental Table 1 — Spikes types by individual mice. [file Table_1.DOCX]

**Supplemental Table 1**. Spikes types by individual mice.

Abbreviations: Genot = genotype, tg = APP/PS1 transgenic, wt = wild-type. Drug: C1 = saline, 1^st^ session, C2 = saline 2^nd^ session, D = ethosuximide, E = levetiracetam. T_tot = total recording time in session (seconds), T_Imm = immobility time in session (s). Spike types: C = cortical spikes, M = cortical/cortico-hippocampal spike with a muscle twitch, CH = cortico-hippocampal spike, SPL = spindle-associated spike, FSPL = fast-spindle associated spike, SWD = spike-wave discharge, CHhp = cortico-hippocampal spike with hyperpolarization, GS = giant spike.

| **Mouse** | **Genot** | **Drug** | **T_tot** | **T_Imm** | **C** | **M** | **CH** | **SPL** | **FSPL** | **SWD** | **CHhp** | **GS** |
| --- | --- | --- | --- | --- | --- | --- | --- | --- | --- | --- | --- | --- |
| 30 | tg | C1 | 10801 | 6429 | 256 | 3 | 40 | 142 | 20 | 97 | 3 | 7 |
| 30 | tg | C2 | 10801 | 7999 | 150 | 1 | 44 | 71 | 30 | 175 | 0 | 6 |
| 30 | tg | D1 | 10801 | 10180 | 112 | 2 | 3 | 541 | 0 | 25 | 0 | 6 |
| 30 | tg | E | 10801 | 5811 | 51 | 0 | 0 | 402 | 0 | 56 | 0 | 1 |
| 31 | tg | C1 | 10800 | 8729 | 151 | 0 | 62 | 71 | 19 | 52 | 4 | 28 |
| 31 | tg | C2 | 10860 | 8337 | 102 | 8 | 33 | 97 | 12 | 42 | 1 | 9 |
| 31 | tg | D1 | 10800 | 8767 | 12 | 0 | 5 | 108 | 0 | 1 | 0 | 1 |
| 31 | tg | E1 | 10800 | 9733 | 21 | 1 | 1 | 178 | 0 | 11 | 0 | 4 |
| 32 | wt | C1 | 10800 | 8421 | 75 | 5 | 79 | 100 | 12 | 9 | 0 | 0 |
| 32 | wt | C2 | 10802 | 9137 | 45 | 7 | 112 | 250 | 23 | 17 | 0 | 2 |
| 32 | wt | D1 | 10801 | 9554 | 90 | 2 | 2 | 408 | 0 | 14 | 0 | 0 |
| 32 | wt | E1 | 10821 | 9718 | 47 | 3 | 5 | 321 | 0 | 18 | 0 | 0 |
| 76 | tg | C1 | 11303 | 10463 | 120 | 11 | 49 | 36 | 10 | 4 | 0 | 4 |
| 76 | tg | C2 | 11154 | 10570 | 86 | 10 | 64 | 79 | 7 | 1 | 0 | 6 |
| 76 | tg | D1 | 11381 | 10983 | 81 | 3 | 4 | 66 | 0 | 3 | 0 | 4 |
| 76 | tg | E1 | 10897 | 10365 | 73 | 4 | 4 | 59 | 0 | 2 | 0 | 1 |
| 77 | wt | C1 | 10801 | 8285 | 5 | 3 | 4 | 2 | 0 | 0 | 0 | 0 |
| 77 | wt | C2 | 10801 | 8547 | 9 | 12 | 5 | 6 | 0 | 0 | 0 | 0 |
| 77 | wt | D1 | 11387 | 9787 | 36 | 12 | 4 | 126 | 0 | 2 | 0 | 0 |
| 77 | wt | E1 | 10804 | 9943 | 13 | 5 | 0 | 99 | 0 | 2 | 0 | 0 |
| 78 | wt | C1 | 10800 | 9041 | 548 | 5 | 89 | 15 | 0 | 0 | 0 | 0 |
| 78 | wt | C2 | 10800 | 8661 | 526 | 7 | 177 | 78 | 16 | 0 | 0 | 0 |
| 78 | wt | D1 | 10957 | 5561 | 81 | 1 | 4 | 102 | 0 | 6 | 0 | 0 |
| 78 | wt | E1 | 10800 | 8667 | 42 | 1 | 1 | 93 | 0 | 10 | 0 | 0 |
| 82 | tg | C1 | 10800 | 8687 | 106 | 48 | 44 | 27 | 4 | 2 | 0 | 11 |
| 82 | tg | C2 | 10809 | 9546 | 106 | 60 | 103 | 36 | 6 | 4 | 0 | 5 |
| 82 | tg | D1 | 10801 | 8168 | 120 | 24 | 10 | 74 | 0 | 5 | 0 | 7 |
| 82 | tg | E1 | 10801 | 10368 | 64 | 41 | 4 | 58 | 0 | 2 | 0 | 1 |
| 83 | wt | C1 | 10802 | 6181 | 10 | 8 | 8 | 1 | 1 | 0 | 0 | 0 |
| 83 | wt | C2 | 10800 | 6012 | 36 | 1 | 2 | 56 | 7 | 2 | 0 | 0 |
| 83 | wt | D1 | 10801 | 9595 | 127 | 7 | 4 | 205 | 0 | 8 | 0 | 1 |
| 83 | wt | E1 | 11727 | 10780 | 88 | 1 | 2 | 263 | 0 | 2 | 0 | 0 |
| 87 | wt | C1 | 11112 | 9463 | 53 | 12 | 45 | 146 | 19 | 19 | 0 | 2 |
| 87 | wt | C2 | 10742 | 8344 | 57 | 5 | 23 | 82 | 5 | 1 | 0 | 1 |
| 87 | wt | D2 | 10801 | 8831 | 30 | 0 | 0 | 90 | 0 | 1 | 0 | 1 |
| 87 | wt | E2 | 10802 | 7072 | 84 | 1 | 0 | 200 | 0 | 4 | 0 | 0 |
|  |  |  |  |  |  |  |  |  |  |  |  |  |
|  |  |  |  |  |  |  |  |  |  |  |  |  |
| 96 | tg | C1 | 12397 | 9486 | 176 | 21 | 195 | 213 | 38 | 369 | 1 | 12 |
| 96 | tg | D2 | 10750 | 8655 | 131 | 28 | 7 | 139 | 0 | 55 | 0 | 5 |
| 96 | tg | E1 | 11176 | 9785 | 276 | 8 | 19 | 341 | 0 | 114 | 0 | 4 |
| 97 | tg | C1 | 10802 | 9243 | 38 | 11 | 35 | 15 | 0 | 0 | 22 | 24 |
| 97 | tg | C2 | 11024 | 9497 | 22 | 6 | 38 | 11 | 2 | 2 | 16 | 12 |
| 97 | tg | D1 | 11400 | 9394 | 110 | 8 | 58 | 122 | 2 | 8 | 17 | 54 |
| 97 | tg | E1 | 10749 | 9211 | 69 | 3 | 25 | 82 | 4 | 1 | 0 | 9 |
| 103 | tg | C1 | 10815 | 8266 | 29 | 31 | 23 | 39 | 3 | 14 | 2 | 17 |
| 103 | tg | C2 | 10801 | 9324 | 32 | 27 | 18 | 53 | 2 | 11 | 0 | 20 |
| 103 | tg | D1 | 10800 | 8622 | 12 | 9 | 3 | 20 | 0 | 0 | 0 | 6 |
| 103 | tg | E1 | 10801 | 9982 | 19 | 22 | 8 | 36 | 0 | 1 | 0 | 5 |
| 108 | wt | C1 | 10904 | 9564 | 20 | 3 | 10 | 2 | 1 | 1 | 0 | 0 |
| 108 | wt | C2 | 11032 | 10169 | 22 | 0 | 12 | 8 | 0 | 0 | 0 | 0 |
| 108 | wt | D1 | 10800 | 6951 | 31 | 2 | 16 | 13 | 0 | 0 | 0 | 0 |
| 108 | wt | E1 | 11459 | 10478 | 45 | 3 | 9 | 23 | 0 | 1 | 0 | 0 |
| 124 | tg | C1 | 10822 | 2879 | 43 | 0 | 0 | 1 | 0 | 240 | 0 | 0 |
| 124 | tg | C2 | 10801 | 4139 | 83 | 1 | 12 | 30 | 1 | 467 | 0 | 0 |
| 124 | tg | D2 | 10800 | 1677 | 27 | 0 | 10 | 22 | 0 | 265 | 0 | 0 |
| 124 | tg | E1 | 12086 | 7412 | 113 | 3 | 37 | 105 | 2 | 163 | 0 | 0 |
